# Biomarkers do not paint the whole picture: The role of clinical expertise and advanced neuroimaging for Alzheimer's disease diagnosis

**DOI:** 10.1177/13872877251328953

**Published:** 2025-03-20

**Authors:** Hadrien M Lalive, Alessandra Griffa, Lorenzo Pini, Olivier Rouaud, Gilles Allali

**Affiliations:** 1 Department of Clinical Neurosciences, Leenaards Memory Center, Lausanne University Hospital and University of Lausanne, Lausanne, Switzerland; 2 Medical Image Processing Laboratory, Neuro-X Institute, École Polytechnique Fédérale De Lausanne (EPFL), Geneva, Switzerland; 3 Department of Neuroscience, University of Padova, Padova, Italy

**Keywords:** age-related memory disorders, Alzheimer's disease, biomarkers, functional magnetic resonance imaging

## Abstract

Accurate diagnosis of Alzheimer's disease (AD) in Memory Clinics remains challenging due to the limited specificity of conventional clinical assessment and structural imaging. The recent commentary by Vyhnalek and colleagues advocates for the incorporation of molecular biomarkers for AD diagnosis in clinical practice. However, this approach only partially captures the complexity of disease expression due to co-pathologies such as limbic-predominant age-related TDP-43 encephalopathy, a mimic of AD. At the era of immunotherapy for AD, clinical expertise remains essential to identify AD from its mimics, especially when both entities co-exist, and may rely on advanced neuroimaging techniques such as brain connectivity.

We have read with great interest the commentary by Vyhnalek and colleagues on a recent study by our group showing similar patterns of brain atrophy in older adults with amnestic syndrome related to Alzheimer's disease (AD) or non-AD mimics.^1^ The authors point out the limitations of conventional diagnosis based on clinical syndromes and structural brain imaging, and advocate for the incorporation of molecular biomarkers for AD diagnosis in clinical practice.^2^ While biomarkers enhance diagnostic accuracy, clinical expertise remains essential for AD diagnosis, and advanced neuroimaging methods such as brain connectivity may further improve precision by providing insights into network-level disruptions that are not captured by biomarkers alone.

Vyhnalek et al. suggest that the limited accuracy of conventional diagnostic approaches and imaging markers justify relying mainly on molecular biomarkers for AD diagnosis. However, this approach partially captures the complexity of disease expression and co-pathologies. The authors note the low to moderate accuracy of traditional AD diagnosis in expert clinical centers^3^ and argue that the limitation of brain atrophy patterns in the differential diagnosis of AD supports the revised criteria for diagnosis and staging of AD recently published by the National Institute on Aging and Alzheimer's Association (NIA-AA) Workgroup's.^2^ Briefly, the 2024 NIA-AA guidelines extend the 2018 research framework to clinical practice, suggesting that abnormal “Core 1” biomarkers including cerebrospinal fluid amyloid-β 42 (Aβ_42_), phosphorylated-tau217 (p-tau), p-tau181, p-tau231, and/or amyloid positron emission tomography alone are sufficient for AD diagnosis even in the absence of clinical symptoms. The 2024 guidelines also integrate the biological severity of AD and the severity of clinical impairment as two separate axes of the AD staging scheme.^4,5^ However, a pilot study evaluating implementation of this staging scheme in a Memory Clinic found frequent discrepancy between clinical and biological stages in AD, with 43.5% of subjects exhibiting discordance and 34.7% presenting worse clinical severity than biologically expected.^6^ Lu et al. attributed this mismatch to lifestyle factors and co-pathologies, reporting lower scores of smoking, alcohol consumption, obesity, neurodegeneration and inflammation in resilient patients, thus highlighting the limitations of the accuracy of biomarkers alone in predicting clinical severity. These findings are corroborated by the INSIGHT-preAD study, which demonstrated that the presence of brain β-amyloidosis does not predict conversion to mild cognitive impairment: Dubois and colleagues interpreted this result as the presence of compensatory mechanisms that preserve brain function.^7^ These findings illustrate the ongoing debate about whether brain amyloid and tau deposition should be viewed as active disease biomarkers that predict progression to symptomatic stages or as biological risk factors that increase the likelihood of developing AD, a conceptual distinction that reflects different perceptions of AD between scientific and lay communities.^8,9^ In their recently published response to the NIA-AA diagnostic framework for AD, the International Working Group (IWG) proposes a distinction between asymptomatic individuals at-risk for AD, who present a non-deterministic yet higher risk than biomarker-negative individuals of developing AD, and cognitively normal persons with presymptomatic AD, whose biomarker profile is indicative of an almost deterministic and high lifetime risk of progression to AD.^10^ The IWG framework offers a different diagnostic approach than the AA, defining asymptomatic individuals at-risk for AD as those with brain amyloidosis either isolated or associated with positive fluid phosphorylated tau biomarkers or tauopathy limited to the medial temporal lobe. Presymptomatic AD is instead defined by the presence of brain amyloidosis with tauopathy extending to neocortical regions, autosomal dominant forms of AD, Down syndrome, or homozygous *APOE* ε4 haplotypes with SORL1 loss of function.^10,11^ In their updated recommendations, the IWG contends that over-reliance on molecular biomarkers may lead to abusive AD diagnosis in individuals at low risk of developing symptoms on account of their protective lifestyle factors, robust compensatory mechanisms, and absence of co-pathologies, and may have undue psychological and societal consequences due to stigma and implications for identity and self-determination.^12,13^

While biomarkers improve the specificity of AD diagnosis, clinical expertise remains critical to account for the influence of common co-pathologies that mimic AD. Memory impairment, especially in the oldest-old individuals, are often multifactorial and involves a combination of AD, cerebrovascular disease, and other neurodegenerative conditions such as primary age-related tauopathy (PART), limbic-predominant age-related TDP-43 encephalopathy (LATE), or hippocampal sclerosis (HS).^5^ These conditions, increasingly common in Memory Clinics, are driven by the aging population.^14,15^ PART, LATE, and HS are responsible for amnestic syndrome even in the absence of comorbidities, and LATE and HS significantly influence symptom onset and progression when present in combination with AD.^14–16^ As AD frequently coexists with other dementia-driving diseases in the elderly, interpreting positive Aβ biomarkers in patients over 75 years old is more challenging than in younger subjects since it is uncertain how comorbidity contribute to symptoms.^17^ However, LATE and HS, that are not included in the NIA-AA's staging scheme due to the lack of clinically validated disease-specific in vivo biomarkers, still requires a multifactorial approach integrating AD biomarkers with clinical judgment.^5^ The importance of clinical presentation in the diagnosis of AD is further highlighted by the current European intersocial recommendations for biomarker-based diagnosis of neurocognitive disorders, which require diagnosis of mild cognitive impairment or moderate dementia (Wave 0) and of a clinical syndrome compatible with AD (Wave 1) before using biomarkers to confirm or refute the diagnostic hypothesis (Wave 2).^18^ This approach reinforces the necessity of combining clinical presentation with biomarkers to account for the influence of co-pathologies, and may help to limit AD diagnosis in patients at low risk of developing symptoms.^19^ To further enhance diagnostic accuracy and overcome these limitations, advanced imaging techniques such as brain functional connectivity (FC) may provide additional insights into network alterations that complement molecular biomarkers and clinical evaluation.

FC holds potential to improve the differential diagnosis of neurodegenerative conditions by disentangling network alterations associated with common proteinopathies such as Lewy body dementia (LBD), frontotemporal dementia (FTD), and other AD mimics.^20^ Using functional neuroimaging techniques such as resting-state functional magnetic resonance imaging, signals across brain areas can be measured over time to estimate correlations between neural networks, which support diverse cognitive functions and have differential vulnerabilities to neurodegenerative conditions as different pathogenic proteins may preferentially target specific networks.^21^ For instance, while typical AD is characterized by default mode network (DMN) hypo-connectivity associated to salience network hyper-connectivity, FTD presents with the inverse spatial pattern.^22^ LBD is characterized by altered FC in attention-related networks.^23^ Similarly, TDP-43 accumulation spreading initially from the amygdala results in a predominant limbic FC breakdown in LATE.^20^ FC alterations are also related to disease severity, suggesting a close match between connectivity patterns with tauopathy and clinical evolution.^24,25^ Individual-level FC levels have been shown to predict the spread of pathological tau to neocortical areas^26^ and could therefore contribute to stratify individuals at-risk for AD as defined by the new IWG recommendations.^10^ Moreover, we have recently demonstrated heterogenous dynamic FC alterations in an Alzheimer's Disease Neuroimaging Initiative (ADNI) cohort, which not only differentiate clinical stages, but also distinct ATN (amyloid-tau-neurodegeneration) profiles despite their clinical manifestations associated with AD.^27^

Although molecular biomarkers of AD, such as amyloid- and tau-PET, have been included in recent FC studies^24,27^ the lack of pathological or biological confirmation of neurodegenerative disorders remains a significant limitation in the field.^25^ This issue is particularly pronounced when investigating proteinopathies for which PET tracers are not available, such as LATE,^20^ FTD-spectrum disorders,^22^ and LBD.^23^ Addressing this gap should be a priority for future research, and public datasets, such as the ADNI, can provide a valuable resource in this regard, offering an increasing wealth of post-mortem data and brain connectivity information for a growing number of individuals.^28^ Additionally, patient compliance during extended acquisition times, along with the need for advanced processing and analysis skills, presents significant challenges to the practical implementation of FC in clinical practice.^29^ Despite these limitations, FC provides insights into network-level disruptions that complement molecular biomarkers and clinical evaluation, and is a promising tool that could address the limitations of current AD diagnostic criteria for detecting common co-pathologies and for prognosticating disease progression in at-risk and presymptomatic AD individuals. It is worth noting that several efforts are currently underway to bring connectomics into both clinical neurology and psychiatry practice,^30,31^ which could also be leveraged in the field of neurodegenerative diseases.

In conclusion, while molecular biomarkers are crucial for AD diagnosis, they must currently be combined with clinical presentation to account for the influence of co-pathologies and to capture the complexity of disease progression. Advanced imaging techniques, such as FC, warrant further investigation to develop novel network biomarkers that may complement molecular biomarkers and clinical evaluation by identifying AD from its mimics and stratifying at-risk and presymptomatic AD individuals (Figure 1).

**Figure 1.**
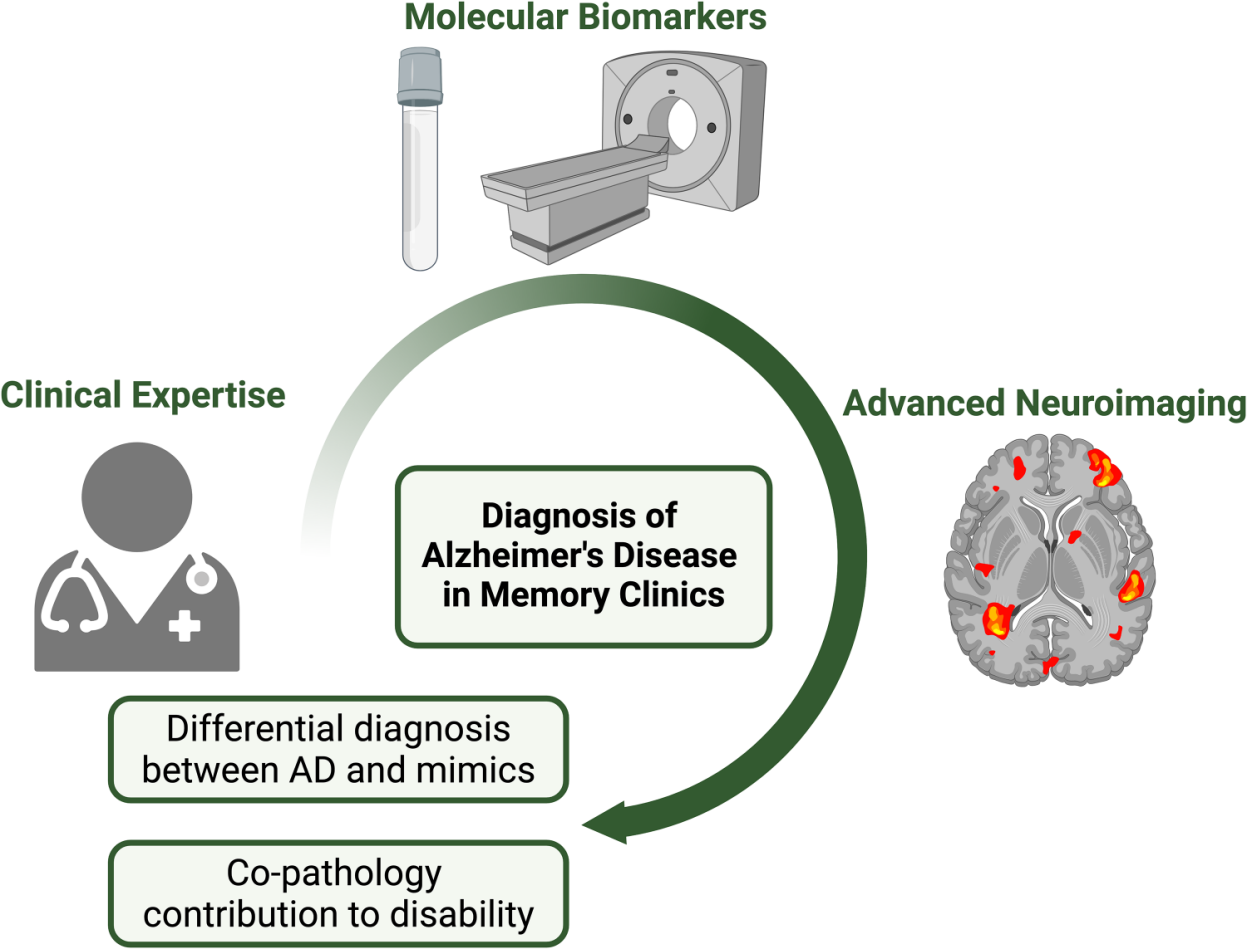
The role of biomarkers, clinical expertise, and advanced neuroimaging in AD differential diagnosis and assessing co-pathologies’ contribution to disability. *Created in BioRender. Lalive, H. (2024) BioRender.com/v99l129*.
